# Innate Immune Activation and Circulating Inflammatory Markers in Preschool Children

**DOI:** 10.3389/fimmu.2021.830049

**Published:** 2022-02-08

**Authors:** Fiona Collier, Cerys Chau, Toby Mansell, Keshav Faye-Chauhan, Peter Vuillermin, Anne-Louise Ponsonby, Richard Saffery, Mimi L. K. Tang, Martin O’Hely, John Carlin, Lawrence E. K. Gray, Siroon Bekkering, David Burgner, the Barwon Infant Study Investigator Group

**Affiliations:** ^1^School of Medicine, Deakin University, Geelong, VIC, Australia; ^2^Child Health Research Unit, Barwon Health, Geelong, VIC, Australia; ^3^Murdoch Children’s Research Institute, Royal Children’s Hospital, Parkville, VIC, Australia; ^4^Department of Neuroepidemiology, The Florey Institute of Neuroscience and Mental Health, Parkville, VIC, Australia; ^5^Department of Paediatrics, Melbourne University, Parkville, VIC, Australia; ^6^Department of Internal Medicine and Radboud Institute for Molecular Life Science (RIMLS), Radboud University Medical Center, Nijmegen, Netherlands; ^7^Department of Paediatrics, Monash University, Clayton, VIC, Australia

**Keywords:** innate immune activation, cytokines, preschool children, human functional genomics, systemic inflammation

## Abstract

Early childhood is characterised by repeated infectious exposures that result in inflammatory responses by the innate immune system. In addition, this inflammatory response to infection is thought to contribute to the epidemiological evidence linking childhood infection and adult non-communicable diseases. Consequently, the relationship between innate immune responses and inflammation during early life may inform prevention of NCDs later in life. In adults, non-genetic host factors such as age, sex, and obesity, strongly impact cytokine production and circulating mediators, but data in children are lacking. Here, we assessed cytokine responses and inflammatory markers in a population of healthy preschool children (mean age 4.2 years). We studied associations between cytokines, plasma inflammatory markers and non-genetic host factors, such as sex, age, adiposity, season, and immune cell composition. Similar to adults, boys had a higher inflammatory response than girls, with IL-12p70 and IL-10 upregulated following TLR stimulation. Adiposity and winter season were associated with increased circulating inflammatory markers but not cytokine production. The inflammatory markers GlycA and hsCRP were positively associated with production of a number of cytokines and may therefore reflect innate immune function and inflammatory potential. This dataset will be informative for future prospective studies relating immune parameters to preclinical childhood NCD phenotypes.

## Introduction

Non-communicable diseases (NCDs) result in huge and increasing human and economic costs globally. Development of innovative and effective prevention of NCDs requires better understanding of modifiable pathogenic pathways across the life course (1). NCDs generally manifest in adulthood and disproportionately affect males (2, 3). The end-organ damage manifests as a range of conditions and inflammation is the central pathogenic process (4). The trajectories that lead to chronic inflammation begin in early life (5). Understanding the variation and early determinants of inflammatory responses in healthy children may therefore highlight opportunities for NCD prevention.

Early childhood is characterised by repeated infectious exposures that result in inflammatory responses by the innate immune system (6, 7). The intensity of these responses in part determines the clinical severity of infection and varies markedly between individuals (8). This inflammatory response to infection is thought to contribute to the epidemiological evidence linking childhood infection and adult NCDs (9). Cytokines mediate these inflammatory responses, but to date, investigation of variation in cytokine production capacity in the general population has been limited to adults (10).

The toll-like receptor (TLR) pathway is central to innate immune responses to microbial stimuli (11) and a potential therapeutic target for inflammatory diseases (12). There are extensive data on TLR responses in older children and adults (13, 14), but few analogous data in preschool children, the age group with the highest incidence of infection (15). In particular, it is unclear whether variation in TLR responses with sex and environmental exposures reported in adults (16, 17) is also evident in early childhood. It is also unknown whether inflammatory markers, such as glycoprotein acetyls (GlycA), an NMR-derived marker of cumulative inflammation (18–20) and high sensitivity C-reactive protein (hsCRP) correlate with innate immune responses.

In the current study, we aimed to investigate innate immune responses to two key bacterial TLR ligands (lipopolysaccharide, LPS; and peptidoglycan, PGN) in a cohort of healthy preschool children, and relate these responses to GlycA and hsCRP. We also examined how host and environmental factors (sex, age, adiposity, granulocyte/monocyte proportions, or season) were related to cytokine production and circulating inflammatory markers.

## Methods

### Study Cohort

Children in this study were participants of the Barwon Infant Study (BIS), which recruited pregnant mothers through antenatal clinics at two major hospitals in Geelong (Victoria, Australia) between 2010-2013 (20). The final inception cohort included 1,064 mothers with 1,074 infants (10 twin pairs). Children who visited the BIS for their preschool clinical review (age 3.9-5.6 years) were asked to complete a questionnaire, undergo a skin-prick allergy test (SPT) and provide a blood sample. The cohort used in this study was a selection of subjects that had both a TLR stimulation assay and relevant metadata. Ethics approval for BIS (Project number 10/24) was obtained through the Barwon Health Human Research Ethics Committee (HREC).

### Data Collection

At the preschool clinical review, parents were asked to complete a survey including questions regarding their child’s current wellbeing, health and any recent illness or temperature. Children also had their body weight [measured with bioelectrical impedance analysis (BIA) scale (Tanita, Kewdale, Australia, model BC-420MA)], height [measuring rod stadiometer (Seca, Hamburg, Germany, model no. 213)] and waist circumference (tape measure) recorded. BMI for each participant was calculated by dividing weight by height in metres squared (kg/m^2^). Measurement of body fat were obtained using the BIA scale.

### Blood Sampling and Innate Immune Assays

Blood samples were collected in preservative-free sodium heparinised tubes. To quantify cytokine responses of immune cells under conditions similar to those *in vivo*, and to maximise outputs from small paediatric blood volumes, we measured the production of monocyte-derived cytokines following stimulation of whole blood. Precisely two hours from blood collection, an aliquot of whole blood was diluted 1:2 with RPMI 1640 growth medium and transferred to plate strips that consist of 8 wells (the size of a 96 well plate) each containing 20μL of either RPMI (growth medium), the gram-negative bacterial membrane component, lipopolysaccharide (LPS, 100ng/mL final concentration) or gram-positive bacterial membrane component, peptidoglycan (PGN, 10ug/mL). These represent either unstimulated, TLR4, or TLR2-stimulated conditions respectively. 180μL of diluted blood was added to each well and cells were stimulated for 24 hours at 37°C in 5% CO_2_. To reduce evaporation, additional plate strips containing only water were incubated alongside the experimental strips. Following the 24-hour incubation, the strips were centrifuged to pellet the blood cells, and the supernatant collected in two 50μL aliquots to be stored at -80°C until cytokine analysis.

### Flow Cytometry

Following phlebotomy, a separate aliquot of 100μL whole blood underwent flow cytometry (Becton Dickinson (BD) FACSCanto™) to identify proportions of target cell populations including monocytes and granulocytes, expressed as the percentage of total white blood cells (WBC). WBC were stained with (i) anti-human CD4-FITC, anti-human CD3-PE and anti-human CD45-PerCP; or (ii) anti-human CD14-FITC, anti-human CD16-PE and anti-human HLA-DR-PECy5, before red cell lysis and formalin fixation. Monocytes and granulocytes were discriminated based on side scatter (SSC) and CD45 expression (21). The proportion of activated nonclassical monocytes (CD14^+^/CD16^++^, as % of total monocytes) was also determined by gating to the HLA-DR^+^ monocytes. All antibodies were sourced from BD Biosciences (San Jose, California, US).

### Cytokine Quantification

Cytokines were quantified using the Bio-Rad Bio-Plex Pro™ cytokine assay kit and detection software, with small modifications to manufacturer instructions. Standards were prepared in a dilution series using the standard kit-provided diluent. Frozen samples were thawed and added to plates that contained 8 standards in duplicate (including blank), and inter-plate controls. Manual steps in the assay included addition of magnetic beads, detection antibodies and streptavidin-phycoerythrin dye, with washing and incubation in between each step. Plates were analysed using the xPONENT MAGPIX ® instrument (Bio-Rad ®). Standard curves were produced based on the standard dilutions and optimised for a recovery rate of 70-130%. Final measures in pg/mL for IL-1β, IL-6, TNFα, IL-12p70 (pro-inflammatory), IL-1RA and IL-10 (anti-inflammatory) were derived by the Bio-Plex Manager™ Software using the standard curves. Cytokine concentrations above the standard curve’s upper limit of quantification (ULOQ) were excluded whereas cytokine concentrations below the lower level of quantification (LLOQ) were replaced with a value equal to 50% of LLOQ.

### GlycA and hsCRP Quantification

Markers of inflammation (GlycA and hsCRP) were measured in plasma samples from the bloods collected in sodium heparin tubes. High-throughput proton NMR metabolomics (Nightingale Health, Helsinki, Finland) quantified GlycA (mmol/L) (22), and hsCRP (ug/ml) was determined using ELISA Human C-Reactive Protein/CRP assay (R&D systems, DY1707). Both measures were log-transformed (base 10), and CRP measurements equal to zero (n=47) were assigned a value equal to 50% of the lowest measure (0.001ug/ml).

### Statistical Analysis

Cytokine measures were log_-_transformed (base 10). BMI z-score standardised for age and sex (based on WHO Child Growth Charts (age <5 years) and the WHO Reference 2007 (age >=5 years) was calculated using the *zanthro* function from the *dm0004_1* user-developed Stata package (23). Spearman correlations were used to quantify the magnitude and direction of association between the cytokines with the coefficient (r_s_) given for each pair of cytokines. Univariable linear regression was used to investigate the associations between exposures, including sex, age, adiposity and innate immune cell proportions, and cytokine levels (log transformed) in the three conditions (unstimulated, and following stimulation with LPS and PGN), as well as the inflammatory markers. The p-values are presented unadjusted for multiple comparisons. Directed acyclic graphs (http://www.dagitty.net) were constructed to determine the minimal sufficient adjustment set for estimating the total effect of the inflammatory markers (GlycA or hsCRP) on the stimulated cytokine levels (**Supplementary Figure 4**). Sensitivity analyses were performed by excluding children with hsCRP above 5ug/ml, which may be indicative of an active infection (24, 25). All statistical analysis and graphics were completed using *Stata Statistical Software: Release 15* (College Station, TX: StataCorp LLC).

## Results

### Baseline Host and Environmental Characteristics

Complete baseline characteristics of the cohort are described elsewhere (20). We have presented the most important baseline characteristics for this study in **Supplementary Table 1** according to sex. 285 participants were included in this study, with 52% male. The mean age for both sexes was 4.2 years. Bloods were collected across all seasons of the year. Mean weight and fat mass was 17.9kg (range 12.6-34.4kg) and 19.7% (range 10.2-29.0%), respectively. Thirty-three children (14.2%) were classified as overweight (BMI z-score >1SD), and 5 children (1.9%) as obese (BMI z-score >2SD) and boys had slightly higher height, weight, BMI and fat mass. Monocyte proportions, but not granulocytes, were modestly higher in boys (mean 7.1± SD 2.1% compared to 6.5 ± 1.9% in girls) whereas the proportion of activated nonclassical monocytes in boys was lower (median 5.4 (IQR 3.4-8.1)% compared to 6.5 (4.2-10.2)%). hsCRP levels were overall lower in males, however 9 (6.1%) boys and 3 (2.1%) girls had hsCRP levels above 5ug/ml possibly indicative of active infection.

### Circulating Inflammatory Markers (GlycA and hsCRP) Associate With Host- and Environmental Factors

Following log transformation of the inflammatory markers, levels of GlycA were only moderately correlated with hsCRP (r=0.41, **Supplementary Figure 1**). Levels of hsCRP were lower in boys (**Supplementary Table 1**, **Table 1**), and GlycA and hsCRP were both positively associated with BMI z-score (**Table 1**). In addition, levels of both inflammatory markers were higher in winter (**Supplementary Figure 2**, **Table 1**), and were associated with the proportion of granulocytes (**Table 1**), indicating overall increased inflammatory status.

**Table 1:** Associations between inflammatory factors and sex, child age, adiposity (BMI and %fat mass), seasons and innate immune cell proportions.

			GlycA	95% CI	p-value	hsCRP	95%CI	p-value
		Adjusted for	Log10(mmol/L)			Log10(ug/ml)		
**Sex (compared to female)**			-0,01	(-0.02, 0.01)	0,285	-0,33		
**Log10** **(Age, yr)**		Sex	0,03	(-0.21, 0.26)	0,820	-0,30		
**BMI z-score**			0,01	(0.00, 0.02)	**0,010**	0,16		
**Log10** **(%Fat Mass)**		Sex, Age	0,19	(-0.01, 0.39)	0,061	4,52		
** **	Summer		-0,03	(-0.05, -0.02)	**<0.0001**	-0,34		
**Season (compared to Winter)**	Autumn		-0,02	(-0.03, 0.00)	**0,035**	-0,63		
** **	Spring		0,02	(-0.04, 0.00)	**0,017**	-0,38		
** **	Granulocytes (% of WB)		0,14	(0.08, 0.21)	**<0.0001**	3,85		
**Innate Immune Populations**	Log10(Monocytes, % of WB)	Sex, Age	-0,03	(-0.08, 0.02)	0,684	0,24		
** **	Log10 (activated nonclassical Monocytes, % of total monocytes)		0,00	(-0.02, 0.02)	0,730	0,65		

Linear regression analysis, *denotes adjusted for sex. Beta coefficients (units=Log10(mmol/L GlycA or ug/ml hsCRP) per variable) with 95% confidence interval (CI) and p value. The strength of association is denoted by either shades of blue (negative) or red (positive).

### TLR-Stimulation and Monocyte Derived Cytokines

To quantify cytokine responses of immune cells under conditions similar to those *in vivo*, and to maximise outputs from small paediatric blood volumes, we measured the production of monocyte-derived cytokines after stimulation of whole blood, rather than purified cell populations. Cells were stimulated for 24 hours using either gram-negative or gram-positive bacterial ligands.

#### Cytokine Correlations

Pro-inflammatory and anti-inflammatory monocyte-derived cytokines (IL-1β, IL-6, TNFα, IL-12p70, IL-1RA, IL-10) were measured following stimulation of TLR4 (LPS) and TLR2 (PGN) in whole blood (**Supplementary Table 2**). Unstimulated cytokine levels moderately predicted the TLR-stimulated levels (except for IL-1β in response to LPS) (**Supplementary Figure 3**). Overall, intra-individual cytokine production in unstimulated and following stimulation by each TLR ligand were modestly to strongly correlated (r_s_ range 0.23-0.84) (**Supplementary Figure 4**).

#### Associations of Cytokines With Host and Environmental Factors

**Figure 1:**
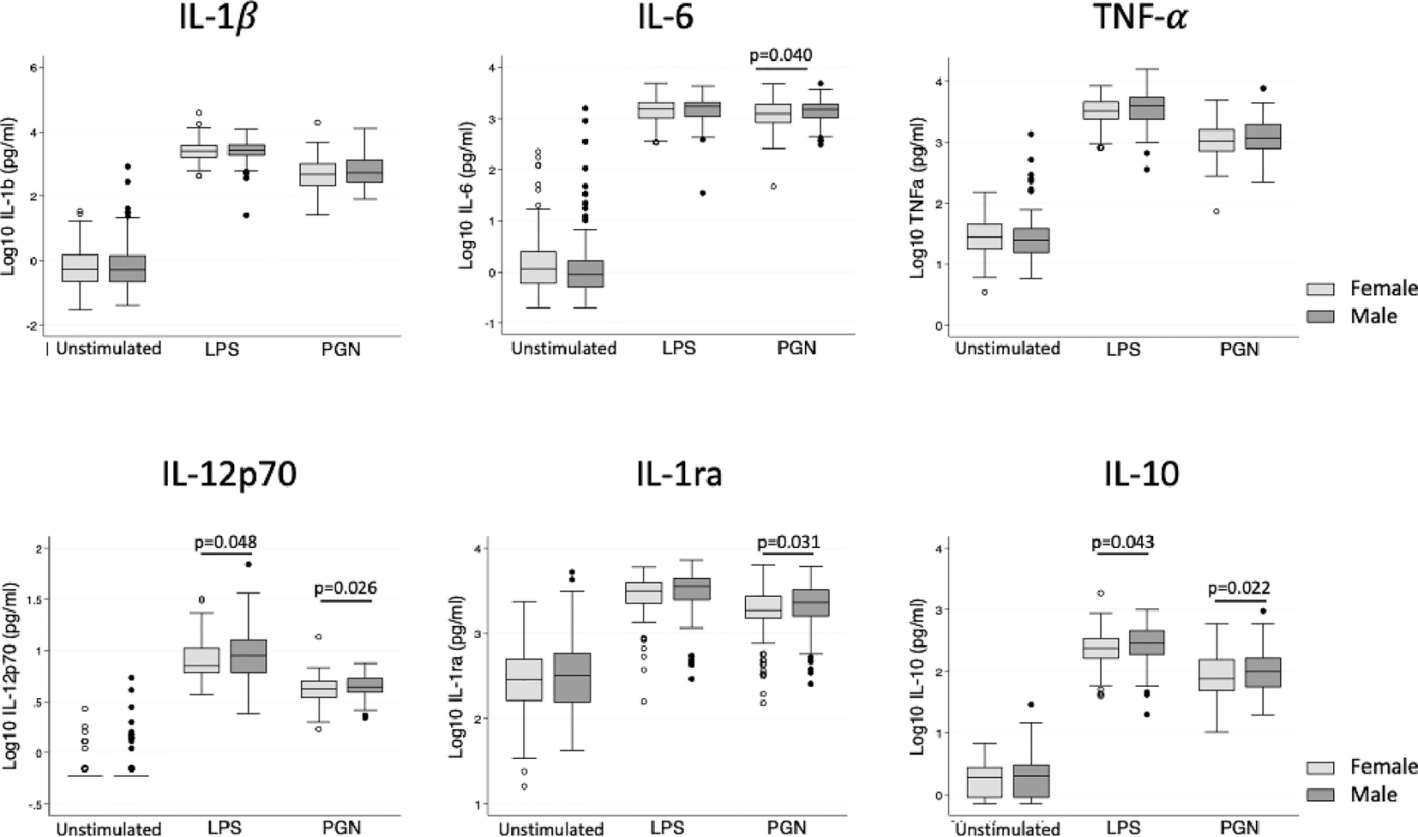
Cytokine levels following *in vitro* stimulation with TLR ligands in female and male children. Box plot graphs (with median and upper and lower quartiles) of cytokine levels (Log_10_(pg/ml)) from whole blood following 24 hour stimulation with medium (unstimulated), LPS (TLR4 stimulation) and PGN (TLR2 stimulation), n=285 (149 males, 136 females). Male children had modestly higher production of IL-12p70 and IL-10 upon both LPS and PGN stimulation and additionally higher IL-6 and IL-1RA upon PGN stimulation (unadjusted linear regression at each treatment).

Both pro- and anti-inflammatory cytokine production following TLR stimulation was higher in boys, particularly for IL-12p70 and IL-10 after LPS stimulation, and IL-6, IL-1RA, IL-12p70 and IL-10 following PGN stimulation (**Figure 1**, **Table 2**). Following adjustment for sex, IL-1β was correlated with age in the unstimulated and LPS stimulated group (**Table 2**). There was no evidence that adiposity was associated with cytokine responses (**Table 2**). Season of blood collection was also not associated with cytokine production (data not shown).

**Table 2:** Association between sex, child age, and adiposity, and log transformed cytokine levels under unstimulated and stimulated conditions.

	Stimulation Conditions	Sex	Log_10_(Age, yr)	BMI z-score	Log_10_(% Fat Mass)
		Compared to Female	Adjusted for Sex		Adjusted for Age and Sex
**IL-1β**	Unstimulated	-0.00 (-0.16, 0.15)	**0.30 (0.02, 0.57)**	0.07 (-0.01, 0.16)	0.01 (-0.01, 0.04)
	LPS	0.00 (-0.08, 0.08)	**-0.18 (-0.33, -0.04)**	-0.03 (-0.07, 0.02)	-0.01 (-0.02, 0.01)
	PGN	0.07 (-0.03, 0.18)	-0.06 (-0.25, 0.13)	-0.00 (-0.03, 0.01)	-0.00 (-0.02, 0.01)
**IL-6**	Unstimulated	-0.07 (-0.22, 0.08)	0.18 (-0.09, 0.44)	0.01 (-0.07, 0.09)	0.00 (-0.02, 0.03)
	LPS	0.02 (-0.03, 0.08)	-0.04 (-0.15, 0.06)	0.01 (-0.02, 0.04)	0.01 (-0.00, 0.02)
	PGN	**0.06 (0.00, 0.12)**	-0.07 (-0.17, 0.04)	0.02 (-0.02, 0.05)	0.00 (-0.01, 0.01)
**TNFα**	Unstimulated	-0.01 (-0.09, 0.07)	0.09 (-0.05, 0.23)	0.01 (-0.04, 0.05)	0.00 (-0.01, 0.02)
	LPS	0.05 (-0.01, 0.11)	0.00 (-0.11, 0.11)	-0.01 (-0.04, 0.02)	0.00 (-0.01, 0.01)
	PGN	0.06 (-0.01, 0.12)	0.00 (-0.12, 0.12)	-0.02 (-0.05, 0.02)	-0.01 (-0.02, 0.01)
**IL-12p70**	Unstimulated	0.02 (-0.01, 0.05)	0.04 (-0.01, 0.09)	0.00 (-0.01, 0.02)	0.00 (-0.00, 0.01)
	LPS	**0.05 (0.00, 0.10)**	-0.02 (0.12, 0.07)	-0.01 (-0.04, 0.02)	0.00 (-0.01, 0.01)
	PGN	**0.03 (0.00, 0.06)**	-0.00 (-0.05, 0.05)	-0.01 (-0.02, 0.01)	-0.00 (-0.01, 0.00)
**IL-1RA**	Unstimulated	0.04 (-0.05, 0.14)	0.13 (-0.02, 0.30)	0.03 (-0.01, 0.08)	0.00 (-0.01, 0.02)
	LPS	0.05 (-0.01, 0.10)	-0.04 (-0.14, 0.07)	0.02 (-0.01, 0.05)	0.01 (-0.00, 0.02)
	PGN	**0.07 (0.01, 0.13)**	-0.01 (-0.12, 0.10)	0.01 (-0.02, 0.04)	-0.00 (-0.01, 0.01)
**IL-10**	Unstimulated	0.02 (-0.05, 0.08)	0.04 (-0.08, 0.16)	0.02 (-0.02, 0.06)	-0.00 (-0.01, 0.01)
	LPS	**0.07 (0.00, 0.14)**	0.04 (-0.08, 0.16)	0.02 (-0.02, 0.05)	0.00 (-0.01, 0.01)
	PGN	**0.09 (0.01, 0.17)**	-0.13 (-0.27, 0.00)	-0.01 (-0.05, 0.03)	0.00 (-0.01, 0.01)

Linear regression analysis. Beta coefficients (units=Log_10_(pg/ml of cytokine) per variable) with 95% confidence interval (CI). Those in bold indicate a p value <0.05

TNFα levels were positively associated with the percentage of monocytes in the stimulated conditions (adjusted for sex, **Supplementary Table 3**). IL-12p70 was associated with the proportion of monocytes in unstimulated blood and following PGN stimulation (**Supplementary Table 3**). IL-1RA was associated with the proportion of granulocytes in unstimulated and TLR-stimulated blood (**Supplementary Table 3**) and there was weaker evidence that IL-6 was associated with granulocyte proportion in unstimulated blood (**Supplementary Table 3**).

### Relationships Between Circulating Inflammatory Markers and TLR-Stimulated Monocyte-Derived Cytokines

**Figure 2:**
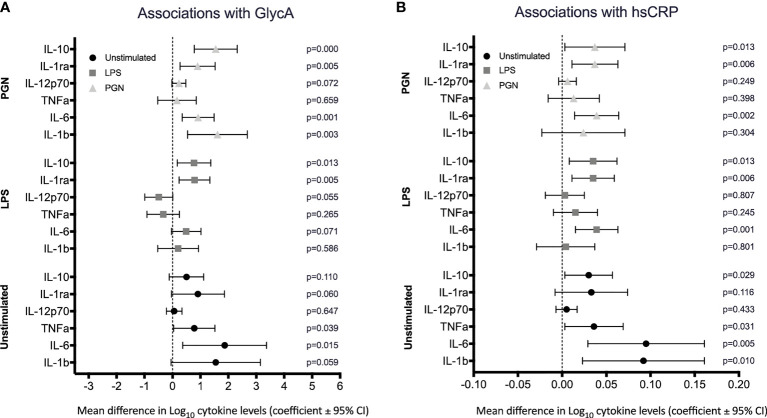
Association between circulating inflammatory markers GlycA and hsCRP and cytokine levels. Cytokines were quantified following stimulation of whole blood in either media (unstimulated) or TLR ligands, LPS and PGN. The estimated mean differences in cytokine levels (Log_10_(pg/ml)) were calculated per 10-fold difference in **(A)** GlycA, Log_10_(mmol/L) and **(B)** hsCRP, Log_10_(µg/ml). Linear regression analyses of cytokine level on inflammatory marker adjusted for sex, age and innate immune cell populations (as determined a priori).

We next investigated associations between circulating inflammatory markers GlycA and CRP and cytokine levels in regression models adjusted for age and sex. There was evidence that both unstimulated and TLR-stimulated cytokine levels were positively associated with GlycA and hsCRP. GlycA was positively associated with the pro-inflammatory cytokines IL-1β and IL-6 in the unstimulated and PGN stimulated group (**Figure 2A**), and with the anti-inflammatory cytokines IL-1RA and IL-10 positively associated with GlycA in the LPS and PGN stimulated groups (**Figure 2A**). High sensitivity CRP was associated with IL-1β and IL-6 in unstimulated blood, with IL-6 following LPS and PGN stimulation (**Figure 2B**), and with IL-1RA and IL-10 following LPS and PGN stimulation (**Figure 2B**).

Sensitivity analyses were performed by excluding children with hsCRP > 5ug/ml, levels possibly indicative of recent or active infection. The associations observed between the circulating inflammatory markers and cytokines in unstimulated and stimulated conditions persisted but were modestly attenuated (**Supplementary Figures 4A, B**).

## Discussion

Here we present the largest study to date investigating the relationships between cytokine responses following TLR stimulation in young children and circulating inflammatory markers. The findings provide novel insights into innate immune activity in healthy preschool children and will inform future studies into early onset of inflammatory diseases in this and other cohorts. Knowledge on healthy innate immune responses provides opportunities for comparison in other cohorts with children or young adults at risk for or with NCDs. Furthermore, the children in our cohort are being followed longitudinally, and further innate immune and CVD related measures are to be performed at 8-10 years of age. In this study, levels of cytokines, both unstimulated and TLR-stimulated, were broadly correlated with each other, and with sex, age and innate immune cell populations, but not with BMI or adiposity measures. Circulating inflammatory markers were associated with cytokine production following TLR stimulation, independent of recent infection.

Infections, which are common in early childhood (15), are associated with increased risk of later NCDs (3, 26, 27). In early childhood, the innate immune responses are central to the defence against infection and are characterised by inflammatory responses (7). Variation in these immune responses in adults is extensively reported (10, 28), but data regarding innate immune capacity and inflammation in preschool children are scarce. In our cohort, prototypical monocyte derived inflammatory cytokines (IL-1β, IL-6 and TNFα) were strongly intercorrelated, particularly in the unstimulated and TLR2-stimulated groups, suggesting co-regulation of cytokine production. This is in keeping with a smaller study of young children (n=57, age 5-96 months), which reported correlations between TNFα and IL-6 in whole blood following LPS stimulation (29). We also found moderate to strong correlations between the anti-inflammatory cytokines, IL-1RA and IL-10, following TLR stimulation, which is consistent with the complex co-regulation of cytokine responses and mechanisms for resolution of inflammation (30, 31).

As NCD prevalence is higher in males, we investigated sex differences in cytokine production in children. In adults, sex differences in cytokine responses are marked (29, 32–34), although this may partly reflect increased monocyte proportions (16). In our preschool cohort, only minor sex differences were evident for some cytokines following TLR stimulation; IL-12p70, IL-10, IL-6 and IL-1RA were modestly increased in boys, who also had higher monocyte proportions. Adjusting for the monocyte proportion attenuated the relationship of male sex and IL-6 and IL-1RA, but not for IL-12p70 and IL-10 (data not shown). In addition, in sex-adjusted models, we found no association between BMI z-score or adiposity and cytokine levels, in contrast to reports from adults (35). This may reflect the relatively small number of children in this study with high BMI z-scores, or the relationship between BMI and innate immune responses may become evident later in life. This is also true for other potential confounders of NCDs, that have not been tested here. Although the adiposity measures were not correlated with innate immune cytokine levels, we noted that both GlycA and hsCRP were positively associated with BMI z-score, in line with similar findings in adults and children (36). Both inflammatory markers were also positively associated with the proportion of granulocytes in these children, consistent with our previous findings from this cohort at 12 months (22) and reflective of generalised inflammatory status. Lastly, we found a correlation between levels of GlycA and CRP and season, which is in line with findings in adults (10).

The association between inflammatory status and cytokine levels in unstimulated and TLR-stimulated blood, which was independent of recent infection, has not previously been reported. As expected, the pro-inflammatory cytokines, IL-1β and IL-6 were most strongly associated with GlycA and hsCRP, particularly in the unstimulated group. This association was not evident following TLR-4 stimulation, and interestingly the fold-change for these cytokines (stimulated/unstimulated) tended to decrease (data not shown); possibly reflecting the association between GlycA and hsCRP with anti-inflammatory cytokines following TLR2 and TLR4 stimulation.

This study has a number of strengths, including the relatively large sample size of young children with data on GlycA and hsCRP levels, as well as detailed cohort meta-data and immune measures. This allowed assessment of potential relationships and adjustment for important confounding variables, including the granulocyte and monocyte proportions, and sensitivity analysis for possible recent infection, which is common in this age group. All experiments were performed on fresh blood within 2 hours of collection, minimising process variation. We also acknowledge some limitations, including the cross-sectional design and the potential for type I errors, given the exploratory nature of the study and the multiple cytokines measured. In addition, it would have been valuable to have complete white blood cell counts, to expand our adjusted assays to lymphocyte counts in addition to monocytes and granulocytes. Although consideration was made for children with hsCRP levels above 5ug/ml, we did not have clinical or microbiological data on recent infections. Finally, future studies in later age groups should also adjust for other metabolic risk factors than BMI, such as lipid profile, glycemia, medication use or auto-immune diseases, which are not clearly apparent at the age of the children in our current study.

In summary, in healthy preschool children, levels of pro- and anti-inflammatory cytokines were correlated before and after TLR2 and TLR4 stimulation of whole blood samples. The inflammatory markers hsCRP and GlycA were positively correlated with innate immune cell activity. The findings support the concept that TLR activation contributes to the development of inflammatory NCD (12). Longitudinal studies from early life, with repeated, standardised measures of innate immune activation and circulating inflammatory markers may inform prediction of later NCD risk and highlight modifiable exposures and opportunities for early prevention.

## Data Availability Statement

The data generated for this study are part of the Barwon Infant Study and are available upon reasonable request. Requests to access the datasets should be directed to https://www.barwoninfantstudy.org.au/.

## Ethics Statement

The studies involving human participants were reviewed and approved by Barwon Health Human Research Ethics Committee (HREC), project number 10/24. Written informed consent to participate in this study was provided by the participants’ legal guardian/next of kin.

## Author Contributions

FC, CC, TM, and KF-C performed acquisition, analysis and interpretation of data. SB and DB contributed to the conceptualization and design of the study. FC, SB, and DB drafted the manuscript. All authors contributed to manuscript revision, read, and approved the submitted version.

## Funding

The initial establishment work and infrastructure for the BIS was supported by the Murdoch Children’s Research Institute and Barwon Health. Deakin University is now a partner organization and has provided funding and infrastructure. Funding for this study was provided by the National Health and Medical Research Council of Australia, NHMRC (1030701). SB is supported by the Dutch Scientific Organisation (452173113) and the Dutch Heart Foundation (2018T028). DB is supported by a NHMRC Investigator Grant (1175744). Research at the Murdoch Children’s Research Institute is supported by the Victorian Government’s Operational Infrastructure Program. The funding bodies did not play any role in the study.

## Conflict of Interest

The authors declare that the research was conducted in the absence of any commercial or financial relationships that could be construed as a potential conflict of interest.

## Publisher’s Note

All claims expressed in this article are solely those of the authors and do not necessarily represent those of their affiliated organizations, or those of the publisher, the editors and the reviewers. Any product that may be evaluated in this article, or claim that may be made by its manufacturer, is not guaranteed or endorsed by the publisher.
