# Supplementary material for: Innate Immune Activation and Circulating Inflammatory Markers in Preschool Children
Source: Front Immunol. 2022 Feb 8;12:830049. doi: 10.3389/fimmu.2021.830049 (PMC8860896; doi:10.3389/fimmu.2021.830049)
Supplement: Supplementary file 1 [file DataSheet_1.docx]

Supplementary Figure 1. GlycA and hsCRP in preschool children. Concentrations of GlycA (Log_10_(mmol/L)) and hsCRP (Log_10_(µg/ml)) in plasma samples were modestly correlated (r=0.410, p<0.0001, n=282).

**Supplementary Figure 2. Seasonal levels of circulating inflammatory factors in preschool children.** There was evidence that A. GlycA, Log_10_(mmol/L) levels were higher in Winter than the other seasons of the year, and B. hsCRP, Log_10_(µg/ml) levels were higher in Winter than in Autumn.

Supplementary Figure 3. Unstimulated cytokine levels generally and moderately predict the TLR-stimulated cytokine levels. Scatter plots and pairwise correlations were used to investigate whether unstimulated cytokine levels (x-axis) predicted the TLR-4 (LPS) and TLR-2 (PGN) stimulated cytokine levels. Levels of cytokines (pg/ml) were Log_10_ transformed.

Supplementary Figure 4. Pairwise correlations between cytokine levels in unstimulated blood and following TLR stimulation. Scatter plots for the cytokines in each stimulatory condition are shown. Levels of cytokines (pg/ml) have been Log_10_ transformed. The respective Spearman’s rank correlation coefficient (r_s_) is shown in each plot. A. Unstimulated; the ‘classic’ inflammatory cytokines, IL-1β, IL-6 and TNFa, were strongly correlated. B. LPS-stimulated; all cytokines were moderately correlated with each other, excluding IL-12p70 with IL-1RA and IL-10. C. PGN stimulated; all cytokines were moderately to strongly correlated with each other, apart from IL-12p70 with IL-1RA.

Supplementary Table 1. Baseline characteristics of the cohort according to sex. All characteristics are listed as mean±SD for normally distributed values, median (with IQR) for non-parametric values, or (%) as listed.

| **Characteristic of Participants (n=285)** | **Male** | **Female** | **p-value** |
| --- | --- | --- | --- |
| Number (%) | 149 (52.3) | 136 (47.7) | 0.79 |
| Age (yr) | 4.2 (4.1-4.3) | 4.2 (4.1-4.3) | 0.72 |
| **Season of blood sample collection, n (%)** |  |  |  |
| Autumn: Mar-May | 43 (28.9%) | 35 (25.7%) | 0.60 |
| Winter: Jun-Aug | 36 (24.2%) | 42 (30.9%) |  |
| Spring: Sep-Nov | 29 (19.5%) | 22 (16.2%) |  |
| Summer: Dec-Feb | 41 (27.5%) | 37 (27.2%) |  |
| **Anthropometry (n=268)** |  |  |  |
| Height (cm) | 106.5 ± 4.4 | 105.8 ± 4.5 | 0.16 |
| Weight (kg) | 18.1 ± 2.4 | 17.7 ± 2.9 | 0.19 |
| BMI z-score | 0.4 ± 1.0 | 0.2 ± 1.0 | 0.06 |
| Fat mass (% of weight)) | 19.9 ± 2.5 | 19.5 ± 3.8 | 0.28 |
| **Innate Immune Cells (n=251)** |  |  |  |
| Granulocytes (% of whole blood) | 53.9 ± 9.6 | 52.9 ± 9.3 | 0.39 |
| Monocytes (% of whole blood) | 7.1 ± 2.1 | 6.5 ± 1.9 | 0.014 |
| Activated nonclassical monocytes (CD14^+^/CD16^++^/HLA-DR^+^, % of monocytes) | 5.4 (3.4-8.1) | 6.5 (4.2-10.2) | 0.014 |
| **Inflammatory Markers (n=282)** |  |  |  |
| GlycA (mmol/L) | 1.11 (1.04 – 1.23) | 1.15 (1.07 – 1.23) | 0.13 |
| hsCRP (ug/ml) | 0.11 (0.02 – 0.73) | 0.22 (0.05 – 0.82) | 0.053 |
| Children with hsCRP >5ug/ml, n (%) | 9 (6.1) | 3 (2.2) | 0.11 |

Differences were tested by either two sample T test (for normally distributed values), Wilcoxon rank sum (for non-parametric values) or chi-squared test (season of collection and children with hsCRP >5ug/ml).

Supplementary Table 2. Cytokine measures following TLR stimulation.

|  | **Cytokine Concentration (pg/ml)** | | | | | | |
| --- | --- | --- | --- | --- | --- | --- | --- |
| **Cytokines** | Unstimulated (n=284) | | LPS (n=275) | | | PGN (n=277) | |
| Pro-inflammatory | Median (with IQR) | Range | Median (with IQR) | Range | Median (with IQR) | | Range |
| IL-1β | 0.5 (0.2-1.5) | 0.0-838.7 | 2683.2 (1778.9-3786.3) | 25.4- 38794.5 | 511.7 (243.7-1126.9) | | 25.8- 19407.7 |
| IL-6 | 1.0 (0.5-2.0) | 0.2-1568.2 | 1590.7 (1065.7-2060.7) | 34.9-4897.1 | 1368.0 (912.8-1890.0) | | 257.3-4723.9 |
| TNFa | 27.2 (16.4-41.4) | 3.5-1363.5 | 3609.7 (2404.9-5087.4) | 360.3-15626.1 | 1102.2 (749.1-1859.3) | | 73.3-7661.8 |
| IL-12p70 | 0.6 (0.6-0.6) | 0.6-2.8 | 7.9 (6.1-11.5) | 2.4-69.3 | 4.4 (3.7-5.1) | | 1.7-13.6 |
| Anti-inflammatory |  |  |  |  |  | |  |
| IL-1RA | 295.5 (161.3-548.1) | 16.1-5193.7 | 3294.8 (2404.3-4267.3) | 158.3-7277.1 | 2064.9 (1564.7-3012.9) | | 152.1-6393.3 |
| IL-10 | 1.9 (0.9-2.8) | 0.7-29.0 | 256.9 (181.4-384.6) | 19.7-1853.9 | 85.3 (52.7-157.0) | | 10.2-953.7 |

Supplementary Table 3. Associations of Immune populations with cytokine levels in unstimulated and stimulated conditions.

|  | **Stimulation Conditions** | **Granulocytes,**  **% of WB** | **Log_10_(Monocytes,**  **% of WB]** | **Log_10_(activated nonclassical monocytes, % of total monocytes)** |
| --- | --- | --- | --- | --- |
|  |  | **Adjusted for sex and age** | | |
| **IL-1β** | Unstimulated | 0.49 (-0.41, 1.39) | 0.46 (-0.19, 1.11) | **0.32 (0.04, 0.59)** |
|  | LPS | -0.14 (-0.59, 0.32) | 0.07 (-0.25, 0.40) | 0.04 (-0.11, 0.18) |
|  | PGN | 0.37(-0.24, 0.97) | 0.38 (-0.05, 0.81) | **0.27 (0.09, 0.45)** |
|  |  | **Adjusted for sex** | | |
| **IL-6** | Unstimulated | **0.86 (0.02, 1.71)** | 0.29 (-0.33, 0.91) | **0.31 (0.06, 0.57)** |
|  | LPS | -0.01 (-0.34, 0.32) | 0.12 (-0.12, 0.36) | 0.00 (-0.11, 0.11) |
|  | PGN | -0.20 (-0.14, 0.54) | 0.16 (-0.09, 0.41) | 0.03 (-0.07, 0.14) |
|  |  |  |  |  |
| **TNFa** | Unstimulated | 0.26 (-0.19, 0.72) | 0.28 (-0.05, 0.60) | 0.11 (-0.03, 0.25) |
|  | LPS | -0.16 (-0.49, 0.17) | **0.29 (0.05, 0.52)** | 0.05 (-0.06, 0.16) |
|  | PGN | -0.01 (-0.39, 0.37) | **0.35 (0.08, 0.62)** | 0.12 (0.01, 0.24) |
|  |  |  |  |  |
| **IL-12p70** | Unstimulated | 0.10 (-0.06, 0.26) | 0.07 (-0.04, 0.19) | 0.03 (-0.02, 0.08) |
|  | LPS | **-0.30 (-0.57, -0.02)** | 0.07 (-0.13, 0.27) | 0.02 (-0.07, 0.12) |
|  | PGN | 0.02 (-0.13, 0.17) | 0.08 (-0.03, 0.18) | 0.00 (-0.04, 0.05) |
|  |  |  |  |  |
| **IL-1RA** | Unstimulated | **1.01 (0.05, 1.53)** | 0.18 (-0.20, 0.56) | 0.13 (-0.04, 0.29) |
|  | LPS | **0.73 (0.42, 1.03)** | -0.10 (-0.33, 0.13) | -0.00 (-0.11, 0.11) |
|  | PGN | **0.96 (0.62, 1.30)** | -0.08 (-0.34, 0.18) | 0.09 (-0.02, 0.20) |
|  |  |  |  |  |
| **IL-10** | Unstimulated | 0.24 (-0.14, 0.61) | -0.01 (-0.29, 0.26) | 0.06 (-0.05, 0.18) |
|  | LPS | 0.09 (-0.28, 0.46) | 0.17 (-0.09, 0.44) | -0.08 (-0.02, 0.04) |
|  | PGN | 0.30 (-0.13, 0.73) | **0.41 (0.10, 0.72)** | 0.11 (-0.02, 0.24) |

Linear regression analysis, beta coefficients (units=Log_10_(pg/ml of cytokine) per Log_10_(% of cell population)) with 95% confidence interval (CI). Beta coefficients in bold indicate p value <0.05.

Supplementary Figure 3. Association between circulating inflammatory markers, GlycA and hsCRP, and cytokine levels in children with hsCRP ≤5ug/ml hsCRP. Cytokines were quantified following stimulation of whole blood with either media (unstimulated) or TLR ligands, LPS and PGN. The estimated mean difference in log10 cytokine levels was calculated per 10-fold difference in A. GlycA, Log_10_(mmol/L) and B. hsCRP, Log_10_(µg/ml) by linear regression analyses of cytokine level on inflammatory marker in only the children that had hsCRP levels ≤5ug/ml adjusted for sex, age and innate immune cell populations (as determined a priori).


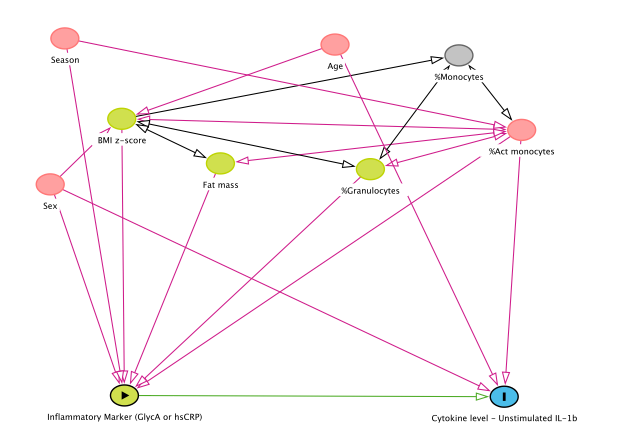


Supplementary Figure 4. Directed Acyclic Graph (DAG) for effects on unstimulated IL-1β levels. In this DAG the inflammatory marker (GlycA or hsCRP) is the exposure, and the cytokine level is the outcome. Relevant covariates were included to develop a network and identify a minimum set of potential confounders to consider in the main analysis. This example estimates the minimal adjustment set for unstimulated IL-1β to be %Act monocytes, age and sex.
